# Regulatory Effects of Cannabidiol on Mitochondrial Functions: A Review

**DOI:** 10.3390/cells10051251

**Published:** 2021-05-19

**Authors:** John Zewen Chan, Robin Elaine Duncan

**Affiliations:** Department of Kinesiology and Health Sciences, Faculty of Health, University of Waterloo, 200 University Ave W, Waterloo, ON N2L 3G1, Canada; l292zhan@uwaterloo.ca

**Keywords:** cannabidiol, oxidative stress, electron transport chain, apoptosis, cannabidiol pharmacokinetics, mitochondrial epigenetics, intracellular calcium, mitochondrial dynamics, mitochondrial biogenesis, mitochondrial ferritin

## Abstract

Cannabidiol (CBD) is part of a group of phytocannabinoids derived from *Cannabis* *sativa.* Initial work on CBD presumed the compound was inactive, but it was later found to exhibit antipsychotic, anti-depressive, anxiolytic, and antiepileptic effects. In recent decades, evidence has indicated a role for CBD in the modulation of mitochondrial processes, including respiration and bioenergetics, mitochondrial DNA epigenetics, intrinsic apoptosis, the regulation of mitochondrial and intracellular calcium concentrations, mitochondrial fission, fusion and biogenesis, and mitochondrial ferritin concentration and mitochondrial monoamine oxidase activity regulation. Despite these advances, current data demonstrate contradictory findings with regard to not only the magnitude of effects mediated by CBD, but also to the direction of effects. For example, there are data indicating that CBD treatment can increase, decrease, or have no significant effect on intrinsic apoptosis. Differences between studies in cell type, cell-specific response to CBD, and, in some cases, dose of CBD may help to explain differences in outcomes. Most studies on CBD and mitochondria have utilized treatment concentrations that exceed the highest recorded plasma concentrations in humans, suggesting that future studies should focus on CBD treatments within a range observed in pharmacokinetic studies. This review focuses on understanding the mechanisms of CBD-mediated regulation of mitochondrial functions, with an emphasis on findings in neural cells and tissues and therapeutic relevance based on human pharmacokinetics.

## 1. Introduction

Cannabidiol (CBD) is a naturally occurring compound that belongs to a group of 142 known phytocannabinoids derived from *Cannabis sativa* [1]. The first documented extraction of pure CBD from a strain of hemp was performed in 1940 [2], and its chemical structure was elucidated roughly 20 years later [3]. Early work determined that, unlike the primary *Cannabis sativa* constituent ∆^9^-tetrahydrocannabinol (THC), CBD is non-psychoactive, and it was presumed to be an inactive compound [4]. As a result, studies on CBD lagged. However, in the 1980s, a plethora of work emerged highlighting its therapeutic potential, and CBD research accelerated [5,6,7,8,9]. Many of these papers focused on the anxiolytic [5], antiepileptic [6,7], anti-convulsive [8], and anti-psychotic [5,9] effects of CBD treatment.

CBD binds with very low affinity to cannabinoid receptor type 1 (CB1) and type 2 (CB2) [10,11]. However, it does not induce marijuana-like effects, but rather acts as an antagonist that counters both endogenous and synthetic CB1/CB2 receptor agonists [12,13]. For example, CBD treatment counters tonic CB1 receptor-mediated signaling in the eye [14], and it also specifically and potently displaces both the mixed CB1/CB2 receptor agonist WIN 55,212 and the synthetic THC mimetic CP 55,940 from brain membranes [12]. Apart from the cannabinoid system, however, CBD also exhibits a wide spectrum of actions in biological systems through interaction with a growing number of known receptors, including the vanilloid receptor 1 (TRPV1) [15], the serotonin 1A receptor (5-HT1A) [16], the peroxisome proliferator-activated receptor gamma (PPARγ) [17], and the adenosine A_2A_ receptor (ADORA2A) [18]. In addition, CBD can have receptor-independent bioactivity in cells, including antioxidant and anti-inflammatory effects [19].

Although these pathways and processes are often investigated separately, they demonstrate notable convergence on mitochondrial function and, indeed, growing evidence has demonstrated a role for CBD in the modulation of mitochondrial processes. Recently, CBD has been found to regulate intracellular and mitochondrial calcium concentrations [20], mitochondria-mediated intrinsic apoptosis [21], mitochondrial DNA epigenetics [22], mitochondrial ferritin concentrations [22], electron transport chain activity [23], mitochondrial biogenesis [24], and mitochondrial network dynamics [25]. Since abnormal mitochondrial function has been closely linked to the development of a wide variety of disorders, ranging from age-related neurodegenerative diseases [26] to cardiovascular diseases [27], diabetes mellitus [28], and multiple cancers [29], development of a more complete understanding of the effects of CBD on mitochondrial function may identify novel therapeutic applications. Here, we review recent advances in the understanding of the mechanisms of CBD-mediated regulation of mitochondrial function, with an emphasis on neural health and translational relevancy based on current knowledge of CBD pharmacokinetics.

## 2. Pharmacokinetics of CBD—A Brief Overview

Miller et al. (2018) compiled the first comprehensive review that focused on pharmacokinetic parameters in humans following CBD administration [30]. They determined that the four main routes of administration of CBD are oromucosal spray, oral ingestion, intravenous administration, and smoking [30]. Not surprisingly, intravenous injection induced the highest recorded plasma concentrations of CBD, with a C_max_ value of 686 ng/mL, or 2.18 μM, measured at 3 min following a 20-milligram administration [31]. This was followed by the smoking of cigarettes containing 19.2 mg of deuterium-label CBD, which caused a spike at 3 min in plasma CBD to 110 ng/mL (0.35 μM) [31]. Oral intake is a common route of administration for CBD. The resulting C_max_ values tends to be lower than those achieved with intravenous administration and are dose dependent. For example, a 10-milligram dose of CBD has been found to result in a C_max_ value of 2.47 ng/mL (or 7.85 nM) at 1.27 h after administration, whereas a dose of 800 mg resulted in a mean C_max_ of 77.9 ng/mL (or 247.7 nM) roughly 3 h post-admission [32,33]. In a more recent study, the pharmacokinetics of oral CBD administration were studied using Epidiolex^®^ in a cohort of patients with Dravet or Lennox–Gastaut syndrome [34]. On average, patients received a dose of 13.2 ± 4.6 mg/kg/day of CBD administered twice daily, which would provide 924 mg/day to a 70-kilogram person. After administration of the morning dose, peak levels 2.5 h later ranged from 0.16 to 2.86 μM, while trough levels 12 h after the evening dose ranged from 32 to 954 nM [34]. Finally, a single dose of an oromucosal spray has been found to result in a C_max_ value between 2.5 and 3.3 ng/mL (7.9 to 10.5 nM) at 1.6 to 4.2 h post-administration [33]. Thus, pharmacologically relevant dosing in humans ranges from the low nanomolar to, at most, a 2–3 μM range. The CBD administration ranges of in vitro studies on mitochondrial metabolism are summarized in Table 1.

## 3. CBD and Mitochondrial Respiration/Bioenergetics

Aerobic cellular metabolism takes place exclusively in the mitochondria of eukaryotic cells [53]. This pathway starts at the tricarboxylic acid cycle (TCA cycle), where nicotinamide adenine dinucleotide (NAD+) and flavin adenine dinucleotide (FAD+) are reduced to yield NADH and FADH_2_ through a series of reactions [53]. Next, complexes I–IV, which form a chain of electron transporters embedded within the inner mitochondrial membrane (IMM), shuttle electrons from NADH and FADH_2_ [53]. Since complexes I, III, and IV are redox-driven proton pumps, electron flow causes protons to be pumped across the IMM into the intermembrane space, generating an electrochemical proton gradient [54]. Proton movement back towards the matrix goes through complex V (ATP synthase) and drives the phosphorylation of adenosine diphosphate (ADP) to create the high energy carrier adenosine-5′-triphosphate (ATP) [55], which is the primary energy source for cells [56]. Thus, abnormal cellular respiration impairs cell function, maintenance, repair, and survival [57].

Specific to the brain, studies on the effects of CBD and the activity of the electron transport chain (ETC) complexes have, to date, been controversial. Valvassori and colleagues (2013) gave adult male Wistar rats either a single intraperitoneal injection or repeated daily injections for 14 days of CBD (60 mg/kg per body weight) [23]. The results indicated that both the acute and repeated administration of CBD elicited a statistically significant increase in complex I, II, III, and IV activity, in both the prefrontal and cerebral tissues of the rat brain [23]. However, this effect was dose dependent, since a decrease in dosage to 15 mg/kg did not elicit a response in the single-dose group and only increased the activity of complexes II and IV in the repeated-dosing group [23]. Although not tested directly, the authors suggested that the likely mechanism behind the effects of CBD on electron transport chain (ETC) activity is an alteration in the regulation of intracellular calcium, either through modulation of the mitochondrial permeability transition pore (mPTP) or the mitochondrial Na^+^/Ca^2+^ exchanger [23]. In the brains of rats treated with CBD, calcium accumulation inside the mitochondria was enhanced, which increased the activity of calcium-sensitive dehydrogenases, promoting the availability of NADH, which enhanced oxidative phosphorylation [23].

Contrary to the previous study, Fisar and colleagues (2014) conducted an in vitro study using isolated pig brain mitochondria to determine the effects of CBD on ETC activity [36]. Interestingly, the application of CBD inhibited the respiration rate of complexes I and II with IC_50_ values of 8.2 ± 0.6 and 19.1 ± 1.1 µM, respectively [36]. The same group of researchers later conducted a follow-up study, which further confirmed the inhibitory effects of complexes I, II/III, and IV after treatment with 50 µM CBD [37]. CBD treatment at this level seemed to exert a greater inhibitory effect on complexes I–III compared to complex IV, with complexes I–III inhibited by approximately three-quarters, but complex IV inhibited by only approximately half [37].

Reports of brain-specific effects of CBD on mitochondrial respiration therefore differ, but had key differences between approaches. Notably, in the first study on intraperitoneal injection of CBD, intact rats were studied, while isolated pig brain mitochondria were used in the second, and differences in the species and mode of administration could have affected outcomes. Differences in effective dosing also likely play a role. In the first study, the blood concentration was not measured. However, based on comparisons to human pharmacokinetic studies, blood concentration was unlikely to have exceeded ~2 μM and was more likely to have been in the nanomolar range [23]. Conversely, in the second study, the lowest concentration of CBD tested was 1 μM [36], while in the third, only 50 μM CBD was investigated [37]. It is possible that these results suggest a biphasic effect of CBD on mitochondrial respiration, where nanomolar concentrations activate, but micromolar concentrations inhibit. A direct comparison of different doses will need to be made in each model to resolve this question. Regardless, the results from the study by Valvassori and colleagues are most likely to reflect actual in vivo conditions, including CBD exposure concentrations during treatment.

In addition to studying brain mitochondria directly, the effects of CBD on respiration have also been tested in a rat primary hippocampal cell culture [20]. In this system, which contained approximately 2:1 neurons to glia, CBD protected against the death of cells subjected to two different mitochondrial toxins that inhibit cellular respiration [20]. First, oligomycin was used to inhibit the phosphorylation of ADP by complex V, which decreased the viability of the cells by 35% [20]. The administration of 1 μM CBD diminished the effects of oligomycin, significantly increasing the number of viable cells by 15 ± 4% [20]. Similar effects were observed following addition of the mitochondrial oxidative phosphorylation uncoupler carbonyl cyanide 4-(trifluoromethoxy)phenylhydrazone (FCCP). While FCCP had a greater negative effect, reducing cell viability by 70%, the co-administration of 1 μM CBD with FCCP once again improved the viability of the cells by 15% [20].

Results from an additional hippocampal study by Sun and colleagues support a protective effect of CBD treatment in the low micromolar range on mitochondrial respiration [41]. In that study, HT22 hippocampal neurons were cultured under conditions of oxygen glucose deprivation to simulate cerebral ischemia, causing cell viability to be reduced by approximately half, while basal respiration was reduced by approximately one quarter. Treatment of cells with 2.5 μM CBD completely restored cell viability, while treatment with 5 μM CBD increased basal respiration in healthy control HT22 cells by greater than 1/3 over vehicle-treated controls and raised basal respiration in ischemic cells to match the healthy control + CBD group.

A role for CBD in the modulation of mitochondrial respiration has been investigated in other tissues. Two papers have examined the effects of CBD on mitochondrial respiration in immunocytes [35,38]. Rimmerman and colleagues (2013) determined that treatment of BV2 murine microglial cells with CBD caused a dose-dependent decrease in mitochondrial membrane potential, with an IC_50_ value of 10 μM [35]. Additional analysis revealed that CBD preferentially colocalized to the mitochondrial voltage-dependent anion channel I (VDAC1), which functions to exchange important metabolites involved in energy metabolism (i.e., ADP and ATP) across the outer mitochondrial membrane (OMM) [35]. Given this role, it suggests that the inhibitory effects of CBD observed in microglial cells were likely mediated, at least in part, by the decrease in conductance of this channel, which, in turn, limited the amount of substrate available for cellular respiration [35].

In a study of THP monocytes, cellular respiration was also determined following treatment with 10.68 and 21.64 μM CBD, concentrations found to inhibit cell viability by 10% and 50%, respectively [38]. While 10.68 μM CBD had no significant effect on mitochondrial bioenergetics, 21.64 μM CBD inhibited the maximal respiration and ATP production of THP monocytes by 58% and 60%, respectively [38]. Current evidence, therefore, indicates that CBD exposure exceeding 10 μM has a detrimental effect on the mitochondrial respiration of immune cells. Additional research is therefore clearly warranted, examining the effects of CBD on mitochondrial respiration of other immune cell types, such as macrophages, B and T cells, dendritic cells, basophils, and others, and also examining CBD doses in a pharmacologically relevant range.

There is significant interest in the potential anti-cancer effects of CBD, and the Lee group investigated the modulation of mitochondrial respiration by CBD in gastrointestinal cancers [39,40]. In cultured AGS gastric cancer cells, the administration of 4 μM CBD caused a significant reduction in both basal respiration rate and overall ATP production [40]. This result was accompanied by a decrease in overall cell proliferation, and decreased tumor growth was observed in vivo when xenografted mice were treated directly with CBD [40]. Similar effects were observed in HCT116 colorectal cancer cells [39], where 6 μM CBD caused a significant decrease in the mitochondrial transmembrane potential [39]. A summary of the effects of CBD on mitochondrial respiration is presented in Table 2.

## 4. CBD and Epigenetic Modifications of Mitochondrial DNA

Epigenetic modifications can alter the expression of genes without changing the DNA sequence [58]. While a majority of cellular DNA is tightly packaged and wrapped around histones within the nucleus, mitochondria contain an isolated subset of cellular DNA as a circular plasmid, making this organelle unique [59]. Mitochondrial DNA (mtDNA) encodes for thirteen different proteins directly involved in oxidative phosphorylation, and it is, thus, critical for cellular respiration [59]. Although it was initially believed that the epigenetic modulation of DNA was exclusive to the nucleus of the cell, recent advances have shown otherwise [60]. This is highlighted in a review by Manev and colleagues (2013), where mtDNA was subjected to epigenetic modulation through the direct intra-mitochondrial processes of methylation and hydroxymethylation [60].

Studies of CBD effects on mitochondrial epigenetics are currently limited in the literature, with only one report published in 2018 by da Silva and colleagues [22]. In this study, the treatment of newborn rats with oral iron for 3 days resulted in significant deficits at 3 months of age in both the methylation and hydroxymethylation of mtDNA in the hippocampal region, with a decrease of more than 80% in 5-methylcytosine (5mC) and of more than 60% in 5-hydroxymethylcytosine (5hmC) content [22]. However, treatment of adult rats at 3 months of age for 14 days with daily intraperitoneal injections of CBD (10 mg/kg) had a profound effect on the reversal of declines caused by neonatal iron, increasing 5mC by over two-fold and restoring 5hmC content to a level that was comparable to controls [22]. Given these findings, the authors suggested that CBD may have an overall protective effect against iron-induced neurodegeneration [22]. Since abnormal mtDNA has previously been linked to the prognosis of various neurogenerative diseases, such as Alzheimer’s disease (AD) and Parkinson’s disease (PD) [61], these findings suggest that further studies are warranted on a role for CBD in the prevention of neurodegeneration through the direct modulation of mtDNA epigenetics.

## 5. CBD and Intrinsic Apoptosis

Apoptosis is a type of programmed cell death that plays a role in many physiological processes [62], ranging from the shaping of embryonic fingers and toes [63] to the elimination of cells invaded by foreign pathogens and to the maintenance and homeostasis of the immune system [62]. The initiation of apoptosis is tightly controlled, and therefore, abnormalities in this process have been shown to play a role in the pathogenesis of neurodegenerative diseases, cancers, viral infections, and other diseases [62]. Two major activating mechanisms control the initiation of apoptosis: the extrinsic pathway mediated by death receptors (i.e., tumor necrosis factor receptors (TNFR), Fas receptor (FasR), and Death receptors 3–5 (DR3-5)) and the intrinsic pathway mediated by the mitochondria [62]. The latter will be the primary focus in this report.

Viruses, DNA damage, UV radiation, cellular and oxidative stress, and growth factor deprivation are all external stressors that can initiate intrinsic apoptosis through the induction of a family of BH3-only proteins (i.e., BAD, BIK, BID, NOXA, etc.) [64,65,66]. This results in the deactivation of BCL-2 anti-apoptotic members, which removes the inhibition of proapoptotic family members (i.e., BAX, BAK, and BOK) [64,65,66]. Active BAX and BAK act on the mitochondria and cause mitochondrial outer membrane permeabilization (MOMP), which promotes the release of cytochrome c and other pro-apoptotic proteins (i.e., SMAC/DIABLO) from the OMM into the cytosol [65,66]. Once in the cytosol, cytochrome c forms a complex with apoptotic protease activating factor 1 (APAF1), which actively cleaves procaspase 9 [37,38,39]. Activated caspase 9 then binds to the APAF1-cytochrome c complex, and altogether, this forms an apoptosome [65]. Subsequently, the apoptosome complex activates caspase 3, which initiates the execution phase of apoptosis through the cleavage of substrates and proteins [62,65]. This induces the typical morphological changes observed in apoptotic cells, including cell shrinkage, nuclear DNA fragmentation, and membrane blebbing [62,65].

The ability of CBD to induce cancer cell apoptosis has been the most highly studied aspect over recent decades [67]. To date, six papers attribute CBD-mediated apoptosis to the intrinsic apoptotic pathway [21,39,40,42,43,44]. Massi and colleagues (2006) were the first group of researchers to highlight the time-dependent induction of intrinsic apoptosis following the administration of 25 μM CBD using a U87 human glioma cancer line [42]. The results from their work demonstrated the increased release of cytochrome c into the cytosol after 10 h of exposure to CBD [42]. This was followed by the maximal activation of caspase 9 approximately fourteen hours later, and caspase 3 seventeen hours later [42]. In addition, CBD also elicited an increase in the formation of reactive oxygen species (ROS) within 5 h of exposure and decreased the total amount of antioxidant glutathione (GSH) within 6 h [42]. Since the rise in ROS and decrease in antioxidant defense occurred prior to the release of cytochrome c, the effect of CBD on the promotion of oxidative stress was likely the initiator of mitochondria-led apoptosis in the glioma cells [42]. Surprisingly, the treatment of healthy primary glial cells with CBD caused no detectable effect on cell viability, even at a concentration of 50 μM [42]. This indicates that CBD preferentially induces the death of cancer cells over normally functioning cells. However, testing of CBD at pharmacologically relevant concentrations is still needed to determine potential therapeutic efficacy in the treatment of gliomas.

Following the first study, McKallip and colleagues (2006) conducted a series of experiments examining CBD and apoptosis in leukemia and lymphoma cell lines [21]. The first experiment showed a dramatic increase in apoptosis of murine EL-4 lymphoma cells grown in vitro following treatment with 2.5 μM CBD [21]. This effect was recapitulated in vivo. C57BL/6 mice were injected intraperitoneally with EL-4 tumor cells and then with 25 mg/kg body weight CBD 10 days later. Twenty-four hours later, cells were collected from the peritoneal cavity, and cancer cell apoptosis was found to be almost 10% greater in mice given the single-dose CBD treatment. In the final experiments, human Jurkat and MOLT-4 leukemia cells were studied. Treatment of cells with 2.5 μM CBD significantly decreased viable cell numbers and increased apoptosis in both lines, with the largest effects evident in MOLT-4 cells. This included a seven-fold decrease in cell viability and an almost 50% increase in the percentage undergoing apoptosis, although effects in Jurkat cells were also significant, with an approximate 33% reduction in cell viability and 15% increase in apoptosis. Further analysis of Jurkat cells revealed greater activation of caspase 9, decreased full-length BID, and increased release of cytochrome c into the cytosol as mechanistic regulators of the observed effects on viability and apoptosis [21]. It was also suggested that the apoptotic effects of CBD on Jurkat cells was due, at least in part, to an increase in oxidative stress mediated by the greater expression of ROS-producing NAD(P)H oxidases [21]. Notably, the concentrations used in this study were aligned with circulating concentrations reported following intravenous infusion of CBD and, therefore, are therapeutically relevant [31].

In a more recent study conducted by Olivas-Aguirre and colleagues (2019), dose-dependent effects of CBD were found [43]. Jurkat lymphoblastic leukemia cell viability was dose-dependently inhibited by CBD (30–100 μM dose range). Effects on proliferation were biphasic depending on the dose, where 1 μM CBD stimulated proliferation while 10 μM CBD had no effect. These results were associated with differences in effects on mitochondrial transmembrane potential. Treatment with 1 μM CBD did not significantly affect the mitochondrial transmembrane potential, while treatment with either 10 or 30 μM CBD significantly dissipated this potential. This was associated with activation of the intrinsic apoptosis pathway due to the release of cytochrome c to the cytoplasm and the induction of caspase 9 and caspase 3 activity. In this study, the primary mechanism of action of CBD was reported to be an increase in the influx of calcium inside the mitochondria, which was predicted to occur through a change in VDAC channel conductance. This change in conductance was hypothesized to be due to direct interaction between CBD and specific residues in the VDAC channel, which would result in the opening of the mPTP and the release of cytochrome c into the cytosol [43]. This study provides strong mechanistic evidence for the regulation of mitochondrial function and intrinsic apoptosis by CBD, albeit at concentrations that are ~4- to 12-fold higher than those achieved by current typical delivery modalities. This work, and in particular, the finding that 1 μM CBD enhanced the proliferation of leukemia cells, highlights the importance of conducting studies using CBD at concentrations that reflect therapeutically attainable blood levels. However, it also highlights the potential clinical benefit of developing novel CBD delivery modes that could achieve higher therapeutic concentrations.

CBD has been found to enhance the apoptotic cell death of two gastric cancer cell lines, AGS and MKN45, with significant effects first observed at 4- and 6-μM treatment levels, respectively [40]. Enhanced apoptosis was reportedly caused by suppression of the anti-apoptotic protein XIAP and enhanced expression of the mitochondrial protein SMAC, which promotes intrinsic apoptosis [40]. Similar observations were also found in HCT-116 and DLD-1 human colorectal cancer cell lines, where treatment with 6 μM CBD increased apoptosis, mediated by an increase in mitochondrial ROS production and the increased expression of the pro-apoptotic protein NOXA [39].

Shrivastava and colleagues studied the effects of CBD on the apoptosis of both estrogen receptor (ER)-positive and -negative human breast cancer cells, including MDA-MB-231, MCF-7, SK-BR-3, and ZR-75-1 [44]. Regardless of ER status, CBD similarly inhibited the proliferation of all lines while also increasing apoptosis in a dose-dependent manner, when amounts of 2.5, 5, 7.5, and 10 μM CBD were tested. However, significant effects were evident only with 5 μM CBD and above. CBD induced typical biomarkers of the intrinsic pathway, including increased activation of caspase 9, increased levels of cleaved beclin-1 and cleaved PARP, and elevated release of cytochrome c. In agreement with previous cancer studies, CBD exposure led to an increase in the generation of ROS, while co-treatment with the antioxidant scavenger alpha-tocopherol significantly diminished the effects of CBD on ROS levels and largely attenuated the CBD-induced apoptosis of breast cancer cells [44]. Of note, CBD administration is followed by increased ROS levels in numerous different cancer cell lines, including breast cancer, gastric cancer, colon cancer, glioma, and leukemia, highlighting that increased oxidative stress is likely a common mechanism by which CBD initiates intrinsic apoptosis in cancer cells [21,39,40,42,44].

Evidence in the literature suggests that CBD can also modify intrinsic apoptosis in the central nervous system [41,45,46]. In a model of hypoxic–ischemic brain injury, forebrain tissue slices from newborn mice were subjected to oxygen glucose deprivation (OGD) and were found to have a five-fold increase in caspase 9 activation [45]. Treatment with a high concentration of CBD (100 μM) attenuated the pro-apoptotic increase in caspase 9 by nearly 60% [45]. It also significantly reduced lactate dehydrogenase efflux, which serves as a marker of cell death [45]. Interestingly, antagonism of the CB2 receptor with AM630 in this study completely abolished the reduction in lactate dehydrogenase efflux, but did not affect caspase 9 activation, indicating likely protective effects of CBD via the CB2 receptor against cell death but not cell apoptosis. Antagonism of the CB1 receptor with SR141716 did not modify the effects of CBD on either cell death or apoptosis, indicating a lack of CB1 receptor involvement.

In a related model, cultured HT22 hippocampal neuronal cells were subjected to oxygen glucose deprivation/reperfusion (OGD/R) and treated in cultures with 5 μM CBD [41]. In this study, CBD profoundly attenuated neuronal cell apoptosis and oxidative stress through the induction of key antioxidants and antioxidant enzymes, including glutathione (GSH), superoxide dismutase (SOD), and glutathione peroxidase (GPX) [41]. In contrast, Mato et al. (2010) found an opposite effect using cultured oligodendrocytes, where brief exposure (i.e., 20–30 min) to CBD at therapeutically relevant levels caused a dose-dependent increase in cell death versus exposure to vehicle alone [46]. Specifically, 100 nM CBD caused an ~10% excess, 1 μM CBD caused an ~15% excess, and 10 μM CBD caused a >30% excess in oligodendrocyte cell death [46]. Interestingly, this effect was not evident in cortical neurons, which did not demonstrate a significant difference in viability with any dose of CBD treatment [46]. In this study, oligodendrotoxicity was associated with increased ROS and hyperpolarization of the mitochondrial membrane [46]. Taken together, these studies indicate that additional work will be needed to elucidate cell-specific effects of CBD, particularly with regard to the major cell types within the central nervous system (e.g., neuroglia, microglia, astrocytes, ependymal cells, and Schwann cells), and likely even within different types of neurons (e.g., hippocampal versus cortical, dopaminergic, glutamatergic, etc.).

Apoptosis is a normal and critical regulatory process for immune system functioning [68], and CBD has been found to alter the intrinsic apoptosis of immune cells. In a study by Wu and colleagues, treatment of human CD14^+^ monocytes with 16 μM CBD induced a greater than 25-fold increase in apoptosis after 1 h, with an approximate doubling evident at 30 min [47]. This was accompanied by a doubling in intracellular ROS at 1 h, but was preceded by a significant increase in the percent of depolarized cells already at 5 min. Cytochrome c release into the cytosol and cardiolipin oxidation were also increased by CBD treatment at this level [47]. To determine the primary mechanism behind the effects of CBD on monocyte apoptosis, the researchers co-administered CBD with a mitochondrial permeability transition pore (mPTP) inhibitor, cyclosporin A (CsA). This treatment markedly attenuated the apoptotic effects of CBD [47], indicating that opening of the mPTP is a mechanism through which CBD induced apoptosis in monocytes in this study.

In another immunological study using murine thymocytes, treatment with 8 μM CBD led to an ~40% increase in the number of apoptotic cells [48]. The co-administration of N-acetyl-L-cysteine (NAC), a precursor of the ROS scavenger glutathione, significantly attenuated the effect of CBD on thymocyte apoptosis, implicating excessive ROS generation in CBD-induced apoptosis of thymocytes [48]. Notably, in both of the studies described, the CBD concentrations utilized were 3- to 7-fold higher than levels that could be obtained by intravenous infusion of CBD and orders-of-magnitude above levels recorded with oral or inhalation-based administration [31,32,33]. The reported effects of CBD on intrinsic apoptosis are summarized in Table 3.

## 6. CBD and Oxidative Stress

Mitochondria play an essential role in the production of energy, which is necessary for cellular survival. However, as a byproduct of energy generation, approximately 1–4% of oxygen reacting with the ETC is only partially reduced, forming reactive oxygen species (ROS) including superoxide anions ($O_{2}^{-}$) [69]. The accumulation of superoxide anions and other ROS, such as hydrogen peroxide and hydroxyl radicals, increases oxidative stress and leads to lipid peroxidation, protein damage, and DNA mutations [69,70]. Mitochondria are particularly susceptible to lipid peroxidation, due to enrichment of the inner mitochondrial membrane with cardiolipin, which has a high content of unsaturated fatty acids [69]. Since cardiolipin is tightly bound to the ETC complexes and is vital to their stability and function, cardiolipin peroxidation can impair energy metabolism. It has also been shown to be an initiator of intrinsic apoptosis [71]. To combat the increase in ROS, the cell has defensive mechanisms to convert ROS into less reactive forms [70,72,73]. For example, manganese superoxide dismutase (MnSOD) converts superoxide anions into less reactive O_2_ and hydrogen peroxide [72]. Subsequently, glutathione peroxidase (GPx) catalyzes the reduction of hydrogen peroxide into O_2_ and water [73]. The abnormal redox balance of ROS is believed to contribute to many pathological disorders [69,74,75,76,77]. Notably, the imbalance between free radicals and antioxidants has previously been shown to be involved in neurodegenerative diseases (e.g., Parkinson’s disease and Alzheimer’s disease), diabetes, atherosclerosis, and cancers [75,76,77,78]. Thus, the utilization of compounds that beneficially target cellular redox balance may have therapeutic potential.

The effects of CBD on oxidative stress and ROS production in neuronal cells are well documented [41,49,50,79,80]. Treatment with 5 µM CBD has been shown to attenuate the OGD/R-induced damage of HT22 hippocampal cells, in part, due to the reduction in oxidative stress [41]. Mechanistically, CBD was found to exert protective effects by attenuating the OGD/R-induced decrease in glutathione content and glutathione peroxidase activity [41]. The neuroprotective effects of CBD have also been studied in a model of primary hippocampal neuronal cells from embryonic Sprague Dawley rats [49]. Co-treatment with 5 µM CBD increased the viability of primary hippocampal neurons treated with 10 µM hydrogen peroxide from 24% to 57% [49]. Despite the observed therapeutic effects, the concentration of CBD treatment alone causing 50% cell death in hippocampal neurons was calculated at 9.85 µM, suggesting that CBD may have a biphasic effect on hippocampal cells, exhibiting neuroprotective effects at lower doses but also causing neurotoxicity at suprapharmacological concentrations [49].

In a neuronal disease model, rat CTX-TNA2 astrocytes were exposed to 300 µM hydrogen peroxide for up to 48 h, which led to increased reactive oxygen species production and apoptosis [50]. Remarkably, co-treatment with 1 µM CBD led to a reduction in ROS production by the 48th hour of treatment and decreased the percentage of cells undergoing early and late apoptosis in response to hydrogen peroxide at both 24- and 48-h time points [50]. The authors tested the intrinsic scavenging/reducing activity of CBD and found that CBD exhibited antiradical effects, and these were comparable to the classic antioxidant butylated hydroxytoluene [50,81]. Thus, it is possible that the decreased ROS production in hydrogen peroxide-treated astrocytes was due to the direct free radical scavenging effects of CBD.

In another neuronal disease model, one- to two-day-old piglets were exposed to hypoxic–ischemic injury (HI) for 30 min by the interruption of carotid blood flow [80]. This increased the oxidative stress of the piglet brain, as indicated by a decrease in GSH/creatine ratio and an increase in protein carbonylation measures [80]. Remarkably, piglets treated intravenously with 1 mg CBD/kg of body weight following the 30 min of HI had a brain activity score that was over three-fold higher than that of piglets infused with the vehicle control solution alone [80]. Subsequent histological analyses found less than half as many necrotic neurons in brains from piglets receiving CBD compared to vehicle, highlighting the neuroprotective role of this compound [80].

The effects of CBD on ROS production by immune cells seem to be dependent on the immune cell types [47,51,52]. For example, 1 µM CBD attenuated the increase in ROS levels in brain microglial cells treated with 10 ng/mL LPS by approximately 70%, while 10 µM CBD completely abolished the ROS-elevating effects of LPS [51]. On the other hand, 8 µM CBD treatment had an opposite effect on splenocytes isolated from male BALB/c mice, increasing oxidative stress by decreasing the content of GSH [52]. Similar effects were also observed in human CD14+ monocytes, where treatment with a high dose of CBD (16 µM) for 1–2 h increased ROS production, which increased cardiolipin peroxidation [47]. Later analysis revealed that the effects of CBD on the ROS production of monocytes was not caused by the modulation of mitochondrial superoxide dismutase (MnSOD), but rather was due to the opening of the mPTP [47].

CBD has been reported to modify ROS levels in diseased cells. As such, a wide variety of CBD doses (2.5–25 µM) have been shown to increase the oxidative stress of many cancer lines [21,39,42,44]. The increase in ROS precedes cancer cell death and is one of the key mechanisms in the anti-cancer effects of CBD [21,39,42,44]. However, in a model of cisplatin-induced nephrotoxicity, intravenous injection with 2.5–10 mg/kg of CBD into C57BL/6J mice for 3 days prevented the elevated kidney ROS levels caused by cisplatin [82]. Additional mechanistic analysis revealed that CBD administration attenuated the increase in the superoxide-generating enzymes NOX4 and NOX1, which led to decreased ROS production, iNOS expression, nitrotyrosine formation, and apoptosis/necrosis of renal tissues with cisplatin damage [82]. These results indicate that caution should be used in combining CBD with chemotherapeutics designed to kill cancer cells through mechanisms involving the induction of oxidative stress.

## 7. CBD and Mitochondrial Regulation of Intracellular Calcium

Ionic calcium is critical for mitochondrial processes including ATP production, which requires a constant supply for proper functioning [83]. Mitochondria play an essential role in the homeostasis of intracellular calcium through the sequestration and release of calcium ions (Ca^2+^) from, and into, the cytosol, and this organelle can act as both a sink and a source for cytosolic calcium [83]. The regulation of Ca^2+^ by mitochondria is therefore important for cell survival, and reports in the literature indicate that CBD can alter the regulation of mitochondrial calcium in multiple different cell lines [20,35,43,46].

Ryan and colleagues (2009) studied Ca^2+^ regulation in primary rat hippocampal cell cultures and in SH-SY5Y human neuroblastoma cells and found that 0.1 and 1 μM CBD mediates bidirectional regulation of Ca^2+^ homeostasis through effects on mitochondria but not endoplasmic reticulum [20]. Notably, the effects differed depending on the cellular excitation state, wherein CBD caused a small rise in intracellular Ca^2+^ concentration in resting neurons but reduced Ca^2+^ oscillations under high-excitability conditions. Using specific inhibitors, it was demonstrated that CBD at these concentrations acts through the mitochondrial Na^+^/Ca^2+^ exchanger (NCX), but not through the mPTP. This differs from a prior report using a higher dose of CBD in human CD14+ monocytes, which demonstrated a functional interaction between CBD and the mPTP [47], and also from a report in human Jurkat cells, where 10 or 30 μM CBD induced a mitochondrial Ca^2+^ overload and mPTP opening [43]. This could indicate differential, concentration-dependent interactions of CBD with various cellular receptors. However, in oligodendrocytes also treated with CBD at doses of 0.1 to 1 μM, a rise in intracellular Ca^2+^ concentrations was recorded that was dependent on mPTP [46]. This also supports the possibility that the effects of CBD may be cell specific as well as concentration dependent.

In BV-2 microglial cells, treatment with either 5 or 10 μM CBD increased intracellular Ca^2+^ concentrations through a direct interaction with the outer mitochondrial membrane protein VDAC1, which markedly reduced channel conductance [35]. These effects were also associated with increased BV-2 microglial cell apoptosis, although 10 μM CBD was 10-fold more effective than 5 μM CBD at inducing a sub-G1 apoptotic cell population, indicating a possible threshold effect of dose.

## 8. CBD and Mitochondrial Fission and Fusion

Mitochondrial fission and fusion processes are vital for the maintenance and proper function of the mitochondrial network [84,85]. During situations of high metabolic stress, fusion causes the extension and interconnection of mitochondria into larger structures [86]. This allows for metabolite, enzyme, and mtDNA exchange throughout a larger mitochondrial network and promotes greater energy production to meet metabolic demands [85]. In addition, fusion can also protect mitochondria damaged by age-related mtDNA mutations, through the fusion of one mitochondrion with mutated DNA to another with healthy, wild-type DNA [84,85,86]. On the other hand, mitochondrial fission involves the breakdown of larger mitochondrial networks into smaller compartments [85]. This process works together with autophagy to allow for the safe removal of damaged parts of individual mitochondria and contributes to the maintenance of a healthy mitochondrial population to promote efficient cellular respiration [84,85].

Currently, only one study has examined the effects of CBD on mitochondrial fission and fusion [25]. In rats overloaded with iron in the neonatal period, decreased levels of Dynamin-1-like protein (DNM1L), a biomarker of mitochondrial fission, were detected in the hippocampus, while decreased levels of Optic atrophy 1 (OPA1), a protein biomarker for mitochondrial fusion, were detected in the cortex [25]. The administration of 10 mg/kg CBD by intraperitoneal injection for 14 days at 3 months of age increased hippocampal DNM1L protein levels comparable to controls, without altering OPA1 protein levels [25]. While further studies will be needed to better understand the effects of CBD on both mitochondrial fission and fusion, this study reveals a promising effect of CBD in the direct modulation of mitochondrial fission.

## 9. CBD and Mitochondrial Biogenesis

The literature on the effects of CBD and mitochondrial biogenesis is limited to a cardiac study by Hao and colleagues [24]. In that work, the results indicated that a single intraperitoneal injection (20 mg/kg) administered to adult male C57Bl/6J mice treated with the potent chemotherapeutic agent doxorubicin (DOX) induced a statistically significant decrease in myocardial mitochondrial copy number, and also in the expression levels of various mitochondrial biogenesis biomarkers, including peroxisome proliferator-activated receptor alpha (PPARα), peroxisome proliferator-activated receptor gamma coactivator 1-alpha (PGC-1α), mitochondrial uncoupling protein 2 (UCP2), mitochondrial uncoupling protein 3 (UCP3), and medium-chain acyl-Coenzyme A dehydrogenase (MCAD), in mouse cardiac samples. Remarkably, intraperitoneal pre-injection of mice with 10 mg/kg CBD 1.5 h prior to injection with DOX attenuated all of the reductions in mitochondria biogenesis biomarkers to levels that were comparable to controls, demonstrating a significant protective effect [24]. Upon further observation, CBD treatment was also found to have had a small positive effect on mitochondrial biogenesis in hearts from mice administered a vehicle control rather than DOX treatment, suggesting that the beneficial effect of CBD on mitochondrial biogenesis is not limited to DOX-treated cells [24]. Since abnormal mitochondrial biogenesis is a component of a variety of metabolic diseases, and also Alzheimer’s disease [87,88], further research is warranted to examine the potential benefits of CBD on mitochondrial biogenesis in other disorders.

## 10. CBD and Mitochondrial Ferritin Regulation

While the expression of cytosolic ferritin is ubiquitous to all tissue types, mitochondrial ferritin (MtF) is expressed exclusively at sites with high metabolic demand, such as the brain, heart, kidney, and testis [89,90,91]. Its primary role involves the storage of free ferrous iron inside the mitochondria, followed by the subsequent conversion of that iron into less reactive forms [89,90]. In a recent in vivo study, neonatal rats were overloaded with iron by intragastric delivery of three daily doses of 30 mg/kg iron carbonyl and were then treated for 14 days at 3 months of age with 10 mg/kg CBD, delivered by intraperitoneal injection, prior to euthanasia [22]. Iron-overloaded rats showed a statistically significant decrease of approximately 50% in the expression level of mitochondrial ferritin in the hippocampal region, which was almost completely reversed by CBD dosing in adulthood [22]. Considering that oxidative damage caused by iron accumulation is evident in specific brain regions of patients suffering from neurodegenerative diseases [92], these data suggest that the regulatory effects of CBD on MtF could have relevance for the design of therapies aimed at reversing neurodegeneration in adults. Nonetheless, more evidence is needed to confirm the modulatory effects of CBD on MtF in other brain regions as well.

## 11. CBD and Monoamine Oxidase

Monoamine oxidases (MAOs) are a family of enzymes embedded onto the outer mitochondrial membrane [93,94]. They catalyze the breakdown of monoamine neurotransmitters, including dopamine, epinephrine, noradrenaline, and serotonin [93,94]. Research on the effects of hashish components on monoamine oxidase concluded that CBD has no effects on the activity of monoamine oxidase in both porcine and human brain tissues [93,95]. However, co-administration of CBD with THC completely diminished the inhibitory effects of THC on monoamine oxidase activity [93]. Although this does not support a direct role for CBD in the modulation of MAO activity, it helps to explain reports of a direct inhibitory effect of CBD on the psychoactive effects of THC [96].

## 12. Concluding Remarks

We have reviewed current knowledge on the multifaceted effects of the phytocannabinoid CBD on mitochondrial functions. This field of research is still at an early stage, although work appears to be accelerating. Comparisons among studies, which would allow for the drawing of broader conclusions, are currently hindered by the wide range of treatment levels and administration modalities employed. While in vivo studies likely bear greater translational relevance for human health, many utilize intraperitoneal injection for administration, which has limitations in drawing direct comparisons with the major routes of human CBD administration—oral, inhalation, and intravenous. Additionally, with only a few exceptions, the majority of the work performed in vitro has utilized CBD at levels exceeding exposures recorded during pharmacokinetic study administration. This suggests that the effects of CBD on the mitochondria could potentially be overestimated in the literature due to the pharmacologically unattainable CBD concentrations that were studied. Alternatively, it is also possible that effects that are relevant to human disease have been missed entirely by current studies employing pharmacologically excessive levels, particularly if CBD has a biphasic effect on cellular processes. Regardless, investigations on the effects of CBD on mitochondria at concentrations that are attainable in humans, as illustrated by pharmacokinetic studies, should be a major focus of future work. In addition, studies should also emphasize broadening the cell and tissue types studied. Evidence of differential regulation by CBD of mitochondrial processes in different cells from the same tissue type—for example, neurons and oligodendrocytes—highlights the critical importance of adding robust data on the molecular effects of CBD in cells. Nevertheless, findings thus far suggest that CBD has biologically relevant effects on mitochondrial functioning that should be further explored in terms of therapeutic efficacy, particularly in the context of use for treatment of mitochondria-related diseases.

## Figures and Tables

**Table 1:** CBD treatment range of all in vitro studies.

Study Title	CBD Treatment	Reference
Direct modulation of the outer mitochondrial membrane channel, voltage-dependent anion channel 1 (VDAC1) by cannabidiol: a novel mechanism for cannabinoid-induced cell death	5 and 10 µM (30 min–6 h)	[35]
Cannabinoid-induced changes in respiration of brain mitochondria	8.2 and 19.1 µM	[36]
Cannabinoid-induced changes in the activity of electron transport chain complexes of brain mitochondria	50 µM (30 min)	[37]
Mitochondrial functions of THP-1 monocytes following exposure to selected natural compounds	5, 7.5, 10, 10.68 15, 20, 21.64, 30, and 40 µM (0–24 h)	[38]
Cannabidiol-induced apoptosis is mediated by activation of Noxa in human colorectal cancer cells	6 µM (0–2 weeks)	[39]
Cannabidiol promotes apoptosis via regulation of XIAP/Smac in gastric cancer	4 and 10 µM (24 h)	[40]
Cannabidiol targets mitochondria to regulate intracellular Ca^2+^ levels	1 µM (overnight, ~12 h)	[20]
Cannabidiol attenuates OGD/R-induced damage by enhancing mitochondrial bioenergetics and modulating glucose metabolism via pentose-phosphate pathway in hippocampal neurons	5 µM (24 h)	[41]
The non-psychoactive cannabidiol triggers caspase activation and oxidative stress in human glioma cells	25 µM (6–24 h)	[42]
Cannabidiol-induced apoptosis in human leukemia cells: a novel role of cannabidiol in the regulation of p22^phox^ and Nox4 expression	2.5 and 5 µM (24 h)	[21]
Cannabidiol directly targets mitochondria and disturbs calcium homeostasis in acute lymphoblastic leukemia	1, 10, 30, 60, and 100 µM (0–72 h)	[43]
Cannabidiol induces programmed cell death in breast cancer cells by coordinating the cross-talk between apoptosis and autophagy	7.5 and 10 µM (12–24 h)	[44]
The neuroprotective effect of cannabidiol in an in vitro model of newborn hypoxic–ischemic brain damage in mice is mediated by CB2 and adenosine receptors	100 µM (30 min)	[45]
Cannabidiol induces intracellular calcium elevation and cytotoxicity in oligodendrocytes	0.1, 1, and 10 µM (0–30 min)	[46]
Cannabidiol induced apoptosis in human monocytes through mitochondrial permeability transition pore-mediated ROS production	16 µM (5 min–2 h)	[47]
A comparative study on cannabidiol-induced apoptosis in murine thymocytes and EL-4 thymoma cells	4, 8, 12, and 16 µM (1–24 h)	[48]
Neuroprotective effects of cannabidiol against hydrogen peroxide in hippocampal neuron culture	1, 5, 10, 15, and 30 µM (24 h)	[49]
Antioxidant and neuroprotective effects induced by cannabidiol and cannabigerol in rat CTX-TNA2 astrocytes and isolated cortexes	1 µM (24–48 h)	[50]
Cannabidiol prevents LPS-induced microglial inflammation by inhibiting ROS/NF-κB-dependent signaling and glucose consumption	1 and 10 µM (30 min–24 h)	[51]
Cannabidiol-induced apoptosis in primary lymphocytes is associated with oxidative stress-dependent activation of caspase-8	1, 2, 4, and 8 µM (15 min–12 h)	[52]

**Table 2:** Effects of CBD on mitochondrial activity.

Tissue Type	CBD Treatment Range	Mitochondrial Respiration	Mechanism of Action	Reference
Prefrontal and cerebral tissue of the rat brain	60 mg/kg/day (14 days) 60 mg/kg (acute) 15 mg/kg/day (14 days) 15 mg/kg (acute)	↑complex I, II, III, and IV activity ↑complex I, II, III, and IV activity ↑complex II and IV activity No effect	↑ intramitochondrial calcium	[23]
Isolated mitochondria from pig brain	IC_50_ = 8.2 ± 0.6 µM IC_50_ = 19.1 ± 1.1 µM (30 min) 50 µM (30 min)	↓complex I activity ↓complex II activity ↓complex I, II/III, and IV activity	DNI	[36,37]
BV2 microglial cells	IC_50_ = 10 µM (1 h)	↓mitochondrial membrane potential	↓ VDAC1 conductance	[35]
THP monocytes	IC_50_ = 21.64 µM (24 h) IC_10_ = 10.68 µM (24 h)	↓maximal respiration ↓ATP production No effect	↓ ETC activity	[38]
OGD/R injured hippocampal cells	5 µM (24 h)	↑succinate dehydrogenase activity ↑ATP production/oxygen consumption rate ↑maximal respiration ↑spare respiratory capacity ↑basal respiration	↑glucose consumption preservation of NADPH/NADP^+^ ratio activiation of glucose-6-phosphate dehydrogenase	[41]
DOX-treated cardiac tissue	10 mg/kg/day IP injection (5 days)	↑complex I and II activity	DNI	[24]
Patient-derived colorectal cancer cells	6 µM (30 min–6 h)	↓mitochondria membrane potential	↑mitochondrial ROS	[39]
AGS gastric adenocarcinoma cells	4 µM (24 h)	↓basal respiration ↓ATP production ↓oxygen consumption	↓expression of NADH dehydrogenase ubiquinone 1α subcomplex subunit 9 (complex I) ↓mitochondrial membrane potential	[40]

DNI = Did not include. ↑ = increase; ↓ = decrease.

**Table 3:** Effects of CBD on intrinsic apoptosis.

Tissue Type	CBD Treatment Range	Intrinsic Apoptosis	Mechanism of Action	Reference
U87 glioma cells	25 µM (6–24 h) 10 µM (6–24 h)	↑apoptosis No effect	↑caspase 9 activation ↑caspase 3 activation ↑cytochrome c release ↑oxidative stress ↓GSH levels No effect	[42]
EL-4 murine lymphoma cells C57BL/6 mice injected with EL-4	1.25 µM (24 h) 2.5 and 5 µM (24 h) 12.5 mg/kg (1 day) 25 mg/kg (1 day)	No effect ↑apoptosis No effect ↑apoptosis	No effect Activation of CB2 No effect DNI	[21]
Jurkat human leukemia cells	2.5 and 5 µM (24 h)	↑apoptosis	Activation of CB2 ↑expression of Nox4 and p22^phox^ ↓mitochondrial membrane potential ↑ROS ↑cytochrome c release ↑activation of caspase 9 ↑activation of caspase 3 ↓full-length BID ↑cleavage of PARP	[21]
Acute lymphoblastic leukemia of T lineage (Jurkat cells)	30 µM (12 h)	↑apoptosis	↓mitochondrial membrane potential ↑caspase 9 activity ↑caspase 3 activity ↑C^2+^ influx into the mitochondria ↑cytochrome c release ↑mPTP opening	[43]
AGS gastric cancer cells	4 µM (24 h)	↑apoptosis	↑ cleaved caspase 9 ↑ cleaved caspase 3 ↑ cleaved PARP ↓XIAP ↑SMAC	[40]
MKN45 gastric cancer cells	10 µM (24 h)	↑apoptosis	↑ cleaved caspase 9 ↑ cleaved caspase 3 ↑ cleaved PARP ↓XIAP ↑SMAC	[40]
Patient-derived colorectal cancer cells	6 µM (12 h–2 weeks)	↑apoptosis	↑ROS ↑NOXA	[39]
MDA-MB-231 breast cancer cells	7.5 and 10 µM (12–24 h)	↑apoptosis	↑ROS ↑cleaved PARP ↑cleaved caspase 9 ↑ cleaved caspase 3 ↑ cleaved caspase 7 ↑t-BID ↑cytochrome c release	[44]
C57BL6 mice forebrain subjected to OGD injury	100 µM (30 min)	↓apoptosis	Interactions with the CB2 and A2a adenosine receptors ↓activation of caspase 9	[45]
HT22 mouse hippocampal cell subjected to OGD/R injury	5 µM (24 h)	↓apoptosis	↓caspase 3 activity ↓PARP activity ↓ROS ↑GSH content ↑SOD activity ↑GPX activity	[41]
Oligodendrocytes isolated from Dawley rat optic nerves	0.1, 1, and 10 µM (20–30 min)	↑apoptosis	↑ROS ↑caspase 9 ↑Ca^2+^ outflux from the mitochondria to the cytosol	[46]
CD14^+^ human monocytes	16 µM (5 min–2 h)	↑apoptosis	↑ ROS ↓MMP ↑mPTP opening	[47]
Thymocytes isolated from male BALB/c mice	4, 8, 12, and 16 µM (4–24 h)	↑apoptosis	↑ROS	[48]
EL4 cells	12 and 16 µM (1–24 h)	↑apoptosis	↑ROS	[48]

DNI = Did not include. ↑ = increase; ↓ = decrease.

## Data Availability

Not applicable.
